# Expression of ATF3 and axonal outgrowth are impaired after delayed nerve repair

**DOI:** 10.1186/1471-2202-9-88

**Published:** 2008-09-18

**Authors:** Harukazu Saito, Lars B Dahlin

**Affiliations:** 1 Department of Orthopaedic Surgery, School of Medicine, Keio University, Tokyo, Japan; 2 Department of Hand Surgery, Malmö University Hospital, Malmö, Sweden

## Abstract

**Background:**

A delay in surgical nerve repair results in impaired nerve function in humans, but mechanisms behind the weakened nerve regeneration are not known. Activating transcription factor 3 (ATF3) increases the intrinsic growth state of injured neurons early after injury, but the role of long-term changes and their relation to axonal outgrowth after a delayed nerve repair are not well understood. ATF3 expression was examined by immunohistochemistry in motor and sensory neurons and in Schwann cells in rat sciatic nerve and related to axonal outgrowth after transection and delayed nerve repair (repair 0, 30, 90 or 180 days post-injury). Expression of the neuronal cell adhesion molecule (NCAM), which is expressed in non-myelinating Schwann cells, was also examined.

**Results:**

The number of neurons and Schwann cells expressing ATF3 declined and the length of axonal outgrowth was impaired if the repair was delayed. The decline was more rapid in motor neurons than in sensory neurons and Schwann cells. Regeneration distances over time correlated to number of ATF3 stained neurons and Schwann cells. Many neurofilament stained axons grew along ATF3 stained Schwann cells. If nerve repair was delayed the majority of Schwann cells in the distal nerve segment stained for NCAM.

**Conclusion:**

Delayed nerve repair impairs nerve regeneration and length of axonal outgrowth correlates to ATF3 expression in both neurons and Schwann cells. Mainly non-myelinating Schwann cells (NCAM stained) are present in distal nerve segments after delayed nerve repair. These data provide a neurobiological basis for the poor outcomes associated with delayed nerve repair. Nerve trunks should, if possible, be promptly repaired.

## Background

Impaired nerve regeneration and weakened target reinnervation are significant clinical problems after nerve injury when the repair of a nerve trunk is delayed [1-3]. Clinically, recovery of motor function is also less than sensory recovery after delayed repair [4], possibly due to the outgrowth of sensory axons interfering with the outgrowth of motor axons [5,6]. In delayed nerve repair the previously injured neurons are reactivated upon the additional injury leading to axonal outgrowth [7]. After a nerve injury Schwann cells are rapidly activated at site of the lesion [8] and in the distal nerve segment with proliferation and remodelling of the extracellular matrix [9]. The insufficient nerve regeneration after delayed nerve repair has been attributed to an inability of Schwann cells to support axonal outgrowth [10,11]. However, detailed signal transduction mechanisms underlying the impaired nerve regeneration are not well understood.

Activating transcription factor 3 (ATF3) is rapidly expressed in neurons and Schwann cells after injury and is preceeded by phosphorylation of c-Jun and JNK activation [12-15]. The number of Schwann cells and neurons in the spinal cord that express ATF3 declines over time if nerve regeneration is prevented [14,16,17], although some sensory neurons may express ATF3 for at least 140 days after injury [16]. A nerve repair 30 days after injury leads to a reduction in the number of ATF3 stained Schwann cells in the distal nerve [14]. However, the consequences of ATF3 expression in neurons and Schwann cells for axonal outgrowth in relation to time after nerve injury are not known, particularly if there is a time limit at which a delayed nerve repair can be performed. In addition, the neural cell adhesion molecule (NCAM) is expressed mainly in non-myelinating Schwann cells [18], but its alteration after delayed nerve repair is unknown. Our aim was to study ATF3 expression in neurons and Schwann cells and relate that to axonal outgrowth and the presence of NCAM, after delayed nerve repair.

## Results

**Figure 1:**
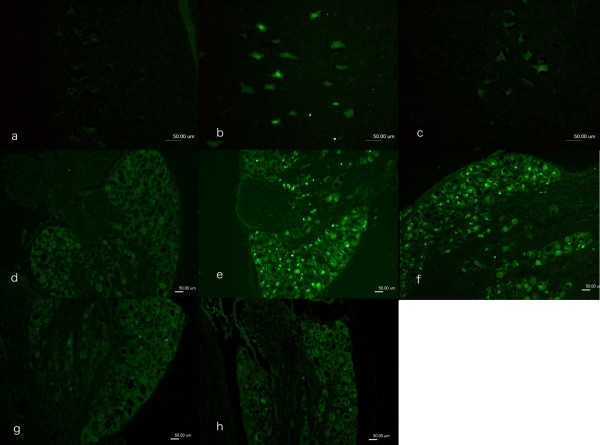
**Immunocytochemical staining of ATF3 in motor neurons in spinal cord (a-c) and in sensory neurons in DRG (d-h)**. In the contralateral side (a, d) no ATF3 stained neurons were observed. ATF3 stained neurons were observed where the sciatic nerve was transected and repaired immediately (day 0, b, e), after 30 days (c, f), after 90 days (g) and after 180 days (h). Scale bar = 50 μm.

**Figure 2:**
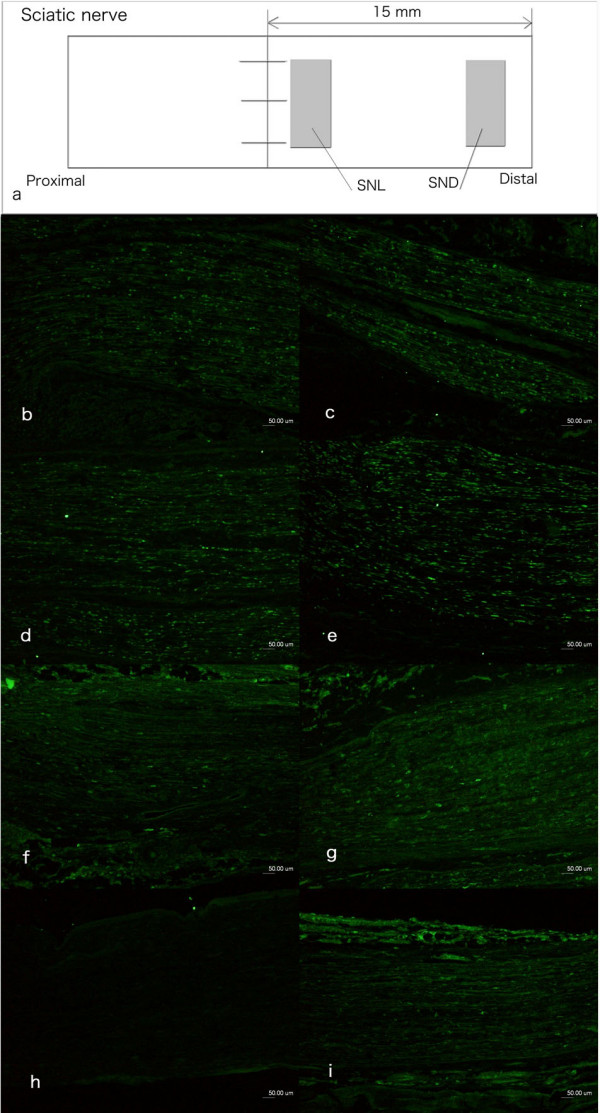
**Immunocytochemical staining of ATF3 in non-neuronal cells in the sciatic nerve.** The non-neuronal cells were interpreted to be mainly Schwann cells based on the shape of the nucleus and location within basal lamina (also stained for S-100). The schematic drawing in (a) showed the sites where ATF3 stained cells were analysed in sciatic nerve of rats. SNL: distal site adjacent to the sutured lesion; SND: 15 mm distal to the sutured lesion. The number of cells was also calculated 15 mm proximal to and at the proximal site of the sutured lesion (results not shown). The photos on the left column (b, d, f) are from the site of lesion (SNL) and in the right column are from the distal nerve segment (SND; c, e, g, i) where the sciatic nerve was immediately repaired (b, c), repair after 30 days (d, e), 90 days (f, g) and 180 days (i). The contralateral uninjured sciatic nerve showed no or single ATF3 stained cells (h). Scale bar = 50 μm.

Nerve transection and repair induced expression of ATF3 in motor and sensory neurons and in non-neuronal cells (i.e. Schwann cells; see below) at the site of the lesion and in the distal nerve segment (Figure 1 and 2). The number of neurons and Schwann cells that stained for ATF3 are summarised in Table 1 [see Additional file 1]. Generally, a significantly (Mann-Whitney; p < 0.005) higher number of ATF3 stained neurons and Schwann cells were present on the experimental side compared to contralateral, non-injured, side at all time points (result not shown) except: 1) number of ATF3 stained Schwann cells in the segment 15 mm proximal to the lesion were not different (result not shown); 2) number of motor neurons in the groups where the sciatic nerve was repaired with a delay of 90 and 180 days. Furthermore, the total number of DAPI stained cells was higher at the experimental sides (distal nerve segment, site of lesion and 15 mm proximal to site of lesion) than in the contralateral nerve at all time points except in the proximal nerve segment (15 mm proximal to suture) after immediate repair.

### Neurons

#### Motor neurons in spinal cord

In the contralateral uninjured side, no ATF3 stained motor neurons were observed (Figure 1a). The number of motor neurons that expressed ATF3 declined over time, where ATF3 stained neurons were mainly observed after immediate repair and to a less number when the sciatic nerve was repaired at 30 days or later (Fig. 1b and 1c). Statistical analyses showed a difference between the groups (Kruskal Wallis; p = 0.006 [see Additional file 1]). Subsequent analyses with the Bonferroni test [19] revealed a statistically significant difference in the number of stained motor neurons in spinal cord between the groups that were repaired immediately (0 days) and the other three groups where a delayed nerve repair was done (repaired 30, 90 and 180 days post injury). However, the latter three groups were not different from each other with respect to number of ATF3 stained motor neurons.

#### Sensory neurons in DRG

In the contralateral side (uninjured) no sensory neurons stained for ATF3 (Figure 1d). Nerve transection and repair induced differences in number of ATF3 stained sensory neurons in DRG between the different groups of immediate and delayed nerve repair (Kruskal Wallis; p = 0.003; Figure 1e–h). There was a statistically (Bonferroni test) higher number of stained neurons after immediate repair and repair 30 days after injury than after a delayed repair at 90 and 180 days after transection [see Additional file 1].

### Non-neuronal cells (i.e. Schwann cells)

The number of non-neuronal Schwann cells was evaluated at four locations (two shown in Figure 2a). The majority of the non-neuronal cells in the proximal (two sites; results not shown) and in the distal nerve segment (SND; Figure 2a) and at the site of the lesions (SNL; Figure 2a) had the characteristics of Schwann cells. They stained for S-100 and were defined as Schwann cells based on morphological characteristics (shape of nucleus and location within basal lamina) in accordance with previous studies [16]. The contralateral uninjured side showed no or only single ATF3 stained cells (Figure 2h).

#### Schwann cells at site of nerve lesion (SND)

There was a significant difference between the nerves repaired at different time points after injury with respect to ATF3 stained Schwann cells just distal to the site of nerve lesion (SNL; Figure 2a; Kruskal Wallis; p = 0.02). The number of ATF3 stained Schwann cells were significantly higher (Bonferroni test [see Additional file 1]) after immediate repair and repair 30 days after transection than in nerves repaired 90 and 180 days after transection (Figure 2b, d, f).

#### Schwann cells in distal nerve segment (SND)

There was a significant difference in ATF3 stained Schwann cells in the nerve segment 15 mm distal to the sutured lesion (SND; Figure 2a; Kruskal Wallis; p = 0.02). The statistical analysis (Bonferroni test) showed significant changes with the same pattern as at the site of nerve lesion (SNL). Thus, after immediate repair (Figure 2c) and repair 30 days post transection (Figure 2e) a significantly higher numbers of ATF3 stained Schwann cells were observed in the distal nerve segment (Bonferroni test [see Additional file 1]) than in the corresponding nerve segment when the nerve trunk was repaired 90 (Figure 2g) and 180 days (Figure 2i) after transection.

#### Total number of DAPI stained cells in distal nerve segment

The total number of DAPI stained cells (not only, but mainly, Schwann cells; S100 staining) in the distal nerve segment (SND; Figure 2a) was higher than in the contralateral control nerve at all time points (Mann Whitney; p < 0.05). There was also a significantly higher total number of DAPI stained cells in the distal nerve segment when repair was done with a delay of 180 days than after immediate (0 days) and a delay of 30 days (Kruskal Wallis; p = 0.016; subsequent Bonferroni test [see Additional file 1]).

### Length of axonal outgrowth

**Figure 3:**
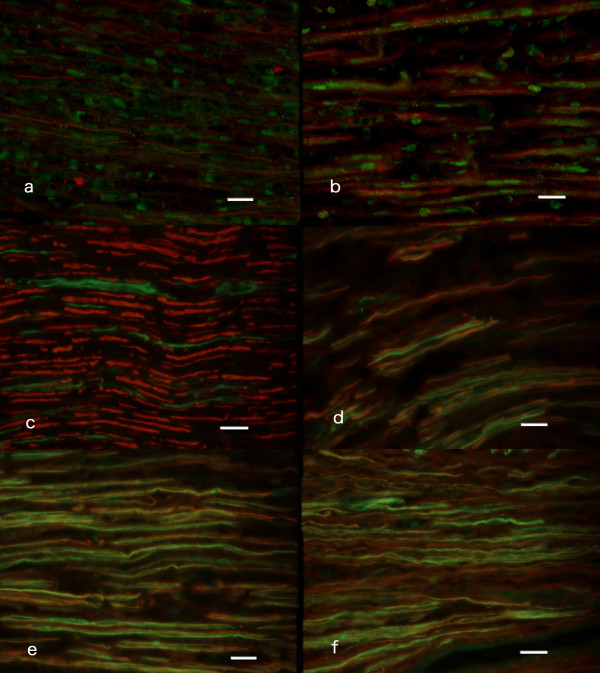
**Images of double labeling of ATF3 and neurofilament (a, b) and NCAM and neurofilaments (c-f).** Double immunochemical staining of ATF3 and neurofilaments (a, b) from the distal nerve after immediate repair (day 0, a) and delayed repair at day 30 (b). Note ATF3 stained Schwann cells close to regenerating fibers. Double immunochemical staining of NCAM (green) and neurofilaments (red) at the contralateral uninjured side (c), at the injured side where sciatic nerve was repaired immediately (d), repaired with delay of 30 days (e) and 90 days (f) after injury. Note the difference in staining of neurofilament and NCAM between c, d, e and f. Scale bar = 50 μm.

The outgrowth length from the site of nerve repair into the distal nerve segment was judged by neurofilament staining. There was a significant difference (Kruskal Wallis p = 0.002 [see Additional file 1]) between the groups of immediate and delayed nerve repair. The outgrowth lengths in nerves repaired immediately (0 days) or 30 days after transection were significantly longer (Bonferroni test [see Additional file 1]) than in nerves repaired 90 and 180 days after transection (no difference between the latter two). Double labelling with anti-ATF3 and anti-neurofilament antibodies showed that axons frequently grow along ATF3 stained Schwann cells (elongated nucleus in basal lamina and stained for S-100; [16]) in the distal nerve segment (Fig. 3a, b).

### Neural cell adhesion molecule (NCAM) in the repaired sciatic nerve

Staining for NCAM was used to visualise non-myelinating Schwann cells [18]. NCAM and neurofilament double-staining were analysed in longitudinal sections both at the site of the lesion and in the distal nerve segment. In contralateral control nerves only patchy areas of NCAM staining were seen and large diameter nerve fibres were not associated with NCAM (Figure 3c). In ten days after transection and immediate repair neurofilament stained fibres with positive NCAM staining had increased, but fibres not associated with NCAM were still seen (Figure 3d). When the repair was delayed 30 (Figure 3e), 90 (Figure 3f) or 180 days after transection almost all regenerating neurofilament stained fibres were associated with NCAM positive staining, although the number of regenerating nerve fibres was reduced at the latter two time points. Thus, the majority of regenerating nerve fibres after delayed nerve repair were associated with non-myelinating Schwann cells (NCAM stained; [18,20]).

### Correlation

The Spearman correlation test revealed a significant correlation between length of axonal outgrowth and the number of ATF3 stained motor neurons (Rho = 0.69, p = 0.005), ATF3 stained sensory neurons (Rho = 0.77, p = 0.002), and in the distal nerve segments [site of lesion (SNL); Rho = 0.76, p = 0.001; nerve segment 15 mm distal to lesion; SND; Rho = 0.71, p = 0.003] when all groups were included. The number of ATF3 stained Schwann cells at the two sites in the proximal nerve segment did not correlate to axonal outgrowth.

The number of ATF3 stained sensory neurons in DRG correlated also to the number of ATF3 stained motor neurons (Rho = 0.68, p = 0.007) and number of ATF3 stained Schwann cells in the distal nerve segment (Rho = 0.71, p = 0.003) when all groups were included. Furthermore, there was a significant correlation between number of ATF3 stained motor neurons and Schwann cells in the distal nerve segment (Rho = 0.48, p = 0.042).

## Discussion

ATF3 is a member of the CREB family and its rapid expression after injury in neurons is preceded by a fast upregulation of JNK and phophorylation of c-jun [12,13,16]. Sensory and motor neurons [13] and non-neuronal cells (i.e. Schwann cells; [14]) in the injured nerve trunk showed a decline in ATF3 response after transection and delayed nerve repair. The reduction of ATF3 stained neurons and Schwann cells correlated to axonal outgrowth. ATF3 expression is long lasting in sensory, but not in motor, neurons if regeneration is prevented [16]. Progression of axonal outgrowth into the distal nerve segment after a nerve crush decreases the number of ATF3 stained motor and sensory neurons both with time and with reinnervation indicating a complex regulation [16]. Retransection of the axons at the time of delayed nerve repair, which is necessary for coaptation, may influence expression of ATF3, since the number of ATF3 expressing neurons was lower than that found when a transection was not repaired [16]. ATF3 expression in Schwann cells decreased with time after denervation in the absence of regeneration [16,17]. In addition, reinnervation of target gives a more rapid decline of ATF3 expression [16]. Interestingly, the number of ATF3 stained neurons and Schwann cells and length of axonal outgrowth correlated when all groups were examined, indicating that delayed nerve repair influences axonal outgrowth and ATF3 expression in neurons and Schwann cells. Therefore, both neurons and Schwann cells are probably of importance for nerve regeneration in delayed nerve repair. Outgrowing axons were associated with ATF3 stained Schwann cells in the distal nerve segment [14] (Figure 3). One may suggest from our and other studies that ATF3 expression in Schwann cells is important for the support of regeneration [14]. As previously shown, ATF3 in neurons promotes neuronal survival and neurite outgrowth, particularly of sensory neurons in DRG [15,21-23]. In addition, inhibition of JNK, and thereby ATF3, reduces outgrowth of sensory axons [12]. We found a correlation between the number of ATF3 expressing neurons and axonal outgrowth. No data are available for a connection between the expression of ATF3 and the regeneration of motor fibers. However, the number of stained motor neurons in the spinal cord correlated with regeneration distances of axons, indicating that not only sensory [21,23], but also motor neurons may be dependent on ATF3 for survival and outgrowth after delayed nerve repair. In the spinal cord, the number of ATF3 stained motor neurons decreased significantly between immediate repair and a repair with a delay of 30 days. Interestingly, the decrease was faster if the nerve was repaired, as in the present study, compared to previous studies when regeneration was not permitted [16], signifying that expression of ATF3 in motor neurons is dependent on interaction between motor neurons and Schwann cells. One may also suggest that early nerve repair is more important for motor neurons than for sensory neurons [24,25], particularly with respect to non-myelinated fibers, as there is better functional motor recovery after early repair [26,27].

The number of ATF3 stained sensory neurons, and the length of outgrowing axons decreased between groups with a repair delay of 30 and 90 days. Neurofilament stained axons were only partly associated with NCAM stained areas of Schwann cells after immediate repair, while after repair at 90 and 180 days post injury almost all regenerating nerve fibers were associated with NCAM stained Schwann cells (Fig. 3d, e, f). The latter findings point towards a more prominent regeneration of non-myelinated axons after a delayed nerve repair, since NCAM (and L1) is associated with non-myelinating Schwann cells and not myelinating Schwann cells [18,20]. However, we cannot differentiate with certainty between sensory and sympathetic axons by the neurofilament staining. Staining for the adhesion molecule L1 has been claimed to be present in Schwann cells of sympathetic unmyelinated axons [20]. In addition, in clinical practice early nerve reconstruction is more effective than delayed reconstruction in relief of pain after brachial plexus injuries [28]; a factor pointing toward a better regeneration of myelinated axons after early repair.

Non-neuronal cells were also stained by ATF3 antibody [13,14], the majority being Schwann cells (characteristics: elongated nucleus, located within basal lamina and S-100 positive) [14,16,29]. Expression of ATF3 in Schwann cells has been seen rapidly after nerve injury [13,14,16,29], but was significantly decreased if repair was delayed for more than 30 days. Although the total number of DAPI stained (mostly, but not only, Schwann cells) cells in the distal nerve segment increased significantly when the nerve was repaired 180 days after injury, compared to the other groups, only around 13% of the Schwann cells stained for ATF3. Double staining of ATF3 stained Schwann cells and neurofilaments in the distal nerve segment (Figure 3) showed a close relationship; but the suggestion that ATF3 may be important for differentiation of Schwann cells, myelination and regeneration [21,30] has to be proven. ATF3 stained Schwann cells were also seen where there were no regenerating fibers in the distal nerve segment, signifying that contact with regenerating nerve fibers is not completely responsible for the decline in ATF3 in Schwann cells [16].

The distal nerve segment is important for axonal outgrowth, as shown particularly well in experiments using short term predegenerated nerve grafts [9]. ATF3 expression in Schwann cells and Schwann cell proliferation, influenced by Erk1/2 expression [8], occurs in the distal nerve segment within a few days of injury [13,14,16,29]. The number of DAPI stained cells (all type of cells; not only, but mostly, Schwann cells) was also higher in the injured side than in the uninjured nerve trunk at all time points. A distal nerve segment denervated for a prolonged period looses its ability to induce and properly support regeneration [5,31,32]. Schwann cells express many regeneration-associated proteins, such as NGFR/p75, GAP43 and extracellular matrix molecules [30,33]. The synthesis of NGFR/p75 in denervated Schwann cells has a similar time course as ATF3 expression with a peak at one month. NGFR/p75 is barely detectable six months after injury [34]. In addition, c-erbB receptors have been investigated in Schwann cells denervated for up to 6 months in vivo [32,35]. The levels of c-erbB receptor expression by Schwann cells, and the degree to which axons regenerated into the distal stumps both decreased with an increased period of denervation. Addition of Leukaemia Inhibitory Factor (LIF) can modulate Schwann cell survival and improve recovery of motor function after delayed nerve repair [36], but a clear connection between ATF3 and LIF has not been established [37,38]. The number of Schwann cells expressing ATF3 decreased with delayed nerve repair and did not increase after repair when compared with previous data [16]. Schwann cells most probably die in the distal nerve segment with delayed nerve repair (see e.g. [31,32]. Thus, nerve repair should be performed as soon as possible to utilise the expression of regeneration associated factors, such as ATF3, in both neurons and Schwann cells.

After nerve injury there is a loss of neurons, particularly in DRG [39-41]. The mechanisms are not completely understood, but ATF3 may determine the cell fate after injury [42-44]. ATF3 as a heterodimer with c-Jun promotes neuronal survival under stress by induction of the anti-apoptotic factor heat shock protein 27 [45]. Inhibition of c-jun activation increases the number of apoptotic sensory neurons after injury in the DRG [22]. Thus, ATF3 plays a role in cell survival and cell cycle. If the present data is compared with previous reports of the decline of ATF3 expression after injury [16], one may suggest that a neuron or a Schwann cell that has lost ATF3 will probably not properly recover their ability to express ATF3 after an additional injury with a diminution of supporting nerve regeneration.

## Conclusion

Delayed nerve repair after an injury impairs the outgrowth of axons in the distal nerve segment. Regeneration distances correlate to the decline of both the number of ATF3 stained neurons and Schwann cells over time. ATF3 stained motor neurons decreased more rapidly than sensory neurons over time. Non-myelinating Schwann cells (NCAM stained) in the distal nerve segment increased when repair was delayed, indicating abundant regeneration of non-myelinated axons after delayed nerve repair. From the neurobiological point of view nerve trunks should, if possible, be promptly repaired.

## Methods

### Animals

Twenty Wistar female rats (weight 180–200 g) were anesthetized, after approval of the local Animal Ethics Committee of Lund University, with an intraperitoneal injection of a mixture of sodium pentobarbital (60 mg/ml, Apoteket AB, Stockholm, Sweden) and sodium chloride (9 mg/ml: 1/10, v/v). Adequate measures were taken to minimize pain and discomfort. Experiments were conducted in accordance with international standards on animal welfare and compliant with local and national regulations.

### Sciatic nerve transection and repair

The sciatic nerve in the left hind limb was exposed and transected 10 mm proximal to the branching of the tibial and peroneal nerves. Four different time points were used to study the influence of delay of nerve repair. In one group the nerve was immediately sutured with 9-0 nylon suture with stitches in the epineurium (day 0; n = 5). In the other groups the distal nerve stump was reversed and put between the muscles at a distance of at least 20 mm from the proximal one to avoid spontaneous reconnection. The wound was closed and the rats were housed in cages. At day 30 (n = 5), 90 (n = 5) or 180 (n = 5) after nerve transection, the rats were re-anesthetized and the nerve ends were sutured with application of 9-0 nylon sutures in the epineurium after retransection of both nerve stumps (resection 0.5–1 mm) to make coaptation easier. The wound was closed and the rats allowed to recover.

Ten days after nerve repair (i.e. 10, 40, 100 and 190 days after the nerves were transected initially) the rats were killed by an i.p. injection with an overdose of sodium pentobarbital followed by a heart puncture. The sciatic nerves bilaterally, the dorsal root ganglia (DRG; L4 and L5) bilaterally and the lumbar enlargement portion of the spinal cord were removed. The samples were fixed in Stefanini fixative (4% paraformaldehyde and 1.9% picric acid in 0.1 M phosphate buffer, pH 7.2) for two hours. They were washed in 0.01 M PBS (phosphate buffered saline, pH 7.4) and kept in 20% sucrose in 0.01 M PBS at 4°C.

### ATF3 expression in the sciatic nerve, spinal cord and the dorsal root ganglia (DRG)

For sectioning the samples were embedded in O.C.T. compound (Sakura Finetek Europe, Leidens, Netherlands) and frozen rapidly in a freezer. The samples were sectioned in a cryostat at 10-μm thickness, and mounted on glass slides. The sciatic nerves were sectioned longitudinally and the spinal cords horizontally. In order to investigate expression of ATF3, the sections were stained with anti-ATF3 antibody (see below).

### Measurement of axonal outgrowth

To measure the length of the regenerating nerve fibers, the sciatic nerve sections (10 μm thick) were stained with anti-neurofilament antibody (see below) and visualized by ABC (Avidin and Biotinylated horseradish peroxidase macromolecular Complex) technique (as detailed below). The slides were photographed using a fluorescence microscope (Eclipse; Nikon, Tokyo, Japan) equipped with a digital camera system (Digital Sight, Nikon, Tokyo, Japan) and the length of axonal outgrowth was measured. Some sections of the sciatic nerves were also double-labelled with the anti-ATF3 antibody and the anti-neurofilament antibody to clarify the relationship between regenerating axons and ATF3 positive Schwann cells.

### NCAM expression in the sciatic nerve

In order to visualize the non-myelinating Schwann cells with the regrowing axons, some sections of the sciatic nerves were double-labelled with the anti-NCAM antibody (see below) and anti-neurofilament antibody according to a previous protocol [18].

### Immunocytochemistry

The sections were washed 10 min with PBS (0.01 M, pH 7.4) and incubated with the primary antibodies (as detailed below) dissolved in 0.25% BSA (bovine serum albumin), 0.25% Triton-X (Packard, Meriden, USA) and in 0.01 M PBS overnight at 4°C. After washing with PBS, the sections were incubated for 1 hour at room temperature with the secondary antibodies (as detailed below) diluted in PBS. After washing with PBS, the sections were mounted with VECTASHIELD^® ^Mounting Medium with DAPI (4',6-diamino-2-phenylindole) (Vector Laboratories, Burlingame, CA, USA).

### ABC technique

The sections of sciatic nerve were washed 5 min with PBS (0.01 M, pH 7.4), and incubated with 0.3% H_2_O_2 _in methanol for 30 minutes at room temperature. After washing with PBS, the sections were incubated with diluted horse serum for 20 minutes at room temperature followed by incubation with the primary antibody (as detailed below) diluted in PBS for 2 hours at room temperature. After further washing with PBS, sections were incubated with the secondary antibody (as detailed below) with rat serum diluted in PBS. The sections were incubated with VECTASTAIN^® ^ABC Reagent (Vector Laboratories, Burlingame, CA, USA) for 30 minutes at room temperature after a further wash in PBS and later incubation with 10 mg 3-Amino-9-ethylcarbazole (AEC) (Sigma-Aldrich, St. Louis, MO) dissolved in 50 ml Na-acetate buffer (0.02 M, pH 5.5) including 1.5 M DMSO (Dimethyl sulfoxide) (Sigma-Aldrich, St. Louis, MO) and 0.3% H_2_O_2_for 20 minutes at room temperature. After a final wash with PBS, the sections were stained with Mayers HTX (Histolab Product, Gothenburg, Sweden) and mounted in glycerin.

### Antibodies

The polyclonal rabbit anti-ATF3 antibody (Santa Cruz Biotechnology, CA, USA) was diluted 1:200. The polyclonal mouse anti-neurofilament antibody (Dako, Glostrup, Denmark) was diluted 1:80. The polyclonal rabbit anti-NCAM antibody (Chemicon, CA, USA) was diluted 1:1000. For immunofluorescence with the anti-ATF3 and anti-NCAM antibodies, Alexa Fluoro 488 conjugated goat anti-rabbit IgG antibody (1:500, Molecular Probes, OR, USA) was used as the secondary antibody. The rhodamine-conjugated anti-mouse IgG (1:500, Cappel, Aurora, OI) was used as the secondary antibody to detect neurofilaments in double immunofluorescence staining. For immunostaining by ABC technique with the anti-neurofilament antibody, biotinylated horse anti-mouse IgG antibody (1:200, Immunkemi, Stockholm, Sweden) was used as the secondary antibody.

### Quantification of number of stained cells

For examination and quantification of ATF3, the slides were photographed using the fluorescence microscope equipped with the digital camera system as mentioned above. Cell counting was performed on digital images acquired as described [16].

#### Spinal cord

Sections of the spinal cord (horizontal section) in every 100 μm were analyzed and five sections were randomly selected from the ventral horn for counting ATF3 stained cells [16]. The number of ATF3 stained cells, expressed in percentage of the total number of all neurons with the same characteristics (motor neurons identified based on position, appearance and size in spinal cord), was detected bilaterally. Unstained cells that were larger than the smallest cell, presenting a nucleus, in each section were counted [16].

#### Dorsal root ganglion (DRG)

Three sections in every DRG (L4 and L5), each 100–200 μm apart, were randomly selected and sensory neurons, irrespective of size, and exhibiting ATF3 immunoreactivity in their nucleus, were calculated in percentage of the number of neurons with a clear nucleus [16].

#### Sciatic nerve

Two sections (100–200 μm apart) from the sciatic nerve were randomly selected and pictures (500 × 400 μm size) were taken. From these two pictures, areas [100 × 100 μm (0.01 mm^2^)] were randomly selected for counting. Areas with epineurium/perineurium, large vessels, scar tissue and other non-neuronal tissues were rejected and only the endoneurial space was analyzed. ATF3 stained cells (i.e. Schwann cells; based on appearance of nucleus and location within basal lamina; also stained in some sections for S-100) and DAPI stained cells (including Schwann cells, perineurial cells, fibroblasts, endothelial cells, inflammatory cells and others) in the randomly selected areas were counted. In the operated side, the cells at the distal site adjacent to the sutured site (SNL) and at the 15 mm distal from sutured site (SND) were quantified (Figure 2a). Cells were also counted at two sites 15 mm proximal to the site of nerve lesion and at the proximal site of the site of repair as well as in the contralateral sciatic nerve (control side).

### Statistical methods

The number of ATF3 stained cells is expressed as median (min-max). Non-parametric methods were used to examine any significant difference between the experimental and control side (Mann-Whitney U-test) and between the experimental side at the four different time points (Kruskal Wallis test with subsequent Bonferroni test; [19]. Spearman correlation test was used to detect any correlations with all animals included. A p-value less than 0.05 is accepted as a significant difference.

## Competing interests

The authors declare that they have no competing interests.

## Authors' contributions

Both authors have contributed equally to the article. Both authors read and approved the final manuscript.

## Supplementary Material

Additional file 1 **ATF stained neurons and Schwann cells, neurofilament stained axons and total number of cells after transection and immediate or delayed nerve repair.** Number of ATF3 stained neurons (% of total number) and Schwann cells (% of total number), neurofilament stained axons (regeneration distance; mm) and total number of cells (DAPI stained) in distal nerve segment after sciatic nerve transection and repair immediately or after a delayed nerve repair (repair 30, 90 and 180 days after injury). Evaluation with immunochemistry was done 10 days after the nerve repair. Click here for file
