# Supplementary material for: Expression of ATF3 and axonal outgrowth are impaired after delayed nerve repair
Source: BMC Neurosci. 2008 Sep 18;9:88. doi: 10.1186/1471-2202-9-88 (PMC2556676; doi:10.1186/1471-2202-9-88)
Supplement: Additional file 1 — ATF stained neurons and Schwann cells, neurofilament stained axons and total number of cells after transection and immediate or delayed nerve repair. Number of ATF3 stained neurons (% of total number) and Schwann cells (% of total number), neurofilament stained axons (regeneration distance; mm) and total number of cells (DAPI stained) in distal nerve segment after sciatic nerve transection and repair immediately or after a delayed nerve repair (repair 30, 90 and 180 days after injury). Evaluation with immunochemistry was done 10 days after the nerve repair. [file 1471-2202-9-88-S1.doc]

|  |  | Time of repair (days) | |  |  |
| --- | --- | --- | --- | --- | --- |
|  | 0 | 30 | 90 | 180 | p-value |
| Motor neurons  (spinal cord) | 83 (71-94)^a^ | 8 (0-12) | 0 (0-11) | 0 (0-0) | 0.006 |
| Sensory neurons (DRG) | 43 (29-60)^b^ | 32 (18-54)^b^ | 6 (4-8) | 2 (1-6) | 0.03 |
| Site of lesion  (SNL) | 44 (34-58)^b^ | 37 (33-77)^b^ | 14 (9-19) | 12 (9-13) | 0.002 |
| Distal nerve segment (SND) | 53 (41-80)^b^ | 50 (41-55)^b^ | 19 (8-25) | 13 (10-20) | 0.002 |
| Regeneration distance  (mm) | 12.1 (8.5-15.6)^b^ | 7.6 (6.6-9.9)^b^ | 3.6 (2.2-6.2) | 2.4 (0.9-3.9) | 0.002 |
| Total number of DAPI stained cells in the distal nerve segment (SND; no/mm^2^)^d^ | 3750 (2500-3750) | 3300 (2800-3550) | 3600 (3200-4800) | 4300 (3900-5350)^c^ | 0.016 |

Values are median (min-max). P-values are calculated with Kruskal Wallis test with subsequent Bonferroni test [19]. No or only single cells stained for ATF3 on the contralateral side. ^a^Significantly different from 30, 90 and 180 days. ^b^Significantly different from 90 and 180 days. ^c^Significantly different from 0 and 30 days. No differences were found between 90 and 180 days in any variables. ^d^In contralateral control nerves the corresponding values were 1000 (200-1900); significantly different from repaired side. The total number of DAPI stained cells did not differ between the groups at the other evaluated sites (just distal to suture line (SNL) and two locations in proximal nerve segments) except in the nerve segment 15 mm proximal to repair site; significantly higher at 180 days compared to 0 days. For explanation of SNL and SND see Figure 2a.
